# Women's (health) work: A population-based, cross-sectional study of gender differences in time spent seeking health care in Malawi

**DOI:** 10.1371/journal.pone.0209586

**Published:** 2018-12-21

**Authors:** Sara Yeatman, Stephanie Chamberlin, Kathryn Dovel

**Affiliations:** 1 Department of Health and Behavioral Sciences, University of Colorado Denver, Denver, Colorado, United States of America; 2 Division of Infectious Diseases, David Geffen School of Medicine, University of California Los Angeles (UCLA), Los Angeles, California, United States of America; 3 Partners in Hope, Lilongwe, Malawi; Tulane University School of Public Health and Tropical Medicine, UNITED STATES

## Abstract

**Background:**

There has been a notable expansion in routine health care in sub-Saharan Africa. While heath care is nominally free in many contexts, the time required to access services reflects an opportunity cost that may be substantial and highly gendered, reflecting the gendered nature of health care guidelines and patterns of use. The time costs of health care use, however, have rarely been systematically assessed at the population-level.

**Methods:**

Data come from the 2015 wave of a population-based cohort study of young adults in southern Malawi during which 1,453 women and 407 men between the ages of 21 and 31 were interviewed. We calculated the time spent seeking health care over a two-month period, disaggregating findings by men, recently-pregnant women, mothers with children under two years old, and “other women”. We then extrapolated the time required for specific services to estimate the time that would be needed for each subpopulation to meet government recommendations for routine health services over the course of a year.

**Results:**

Approximately 60% of women and 22% of men attended at least one health care visit during the preceding two months. Women spent six times as long seeking care as did men (t = -4.414, p<0.001), with an average 6.4 hours seeking care over a two-month period compared to 1 hour for men. In order to meet government recommendations for routine health services, HIV-negative women would need to spend between 19 and 63 hours annually seeking health care compared to only three hours for men. An additional 40 hours would be required of HIV-positive individuals initiating antiretroviral care.

**Conclusions:**

Women in Malawi spend a considerable amount of time seeking routine health care services, while men spend almost none. The substantial time women spend seeking health care exacerbates their time poverty and constrains opportunities for other meaningful activities. At the same time, few health care guidelines pertain to men who thus have little interaction with the health care system. Additional public health strategies such as integration of services for those services frequently used by women and specific guidelines and outreach for men are urgently needed.

## Introduction

Access to and use of routine health care has expanded rapidly in sub-Saharan Africa. Although health care is frequently free in government health facilities, there are numerous costs associated with ostensibly “free” care. These include transportation expenses, the cost of some medicines, and the time required from patients and their caregivers [1–4]. The time it takes to access health services can be substantial in African settings but often falls outside the calculus of policymakers [5]. This gap is concerning because neglecting time expenditures undervalues patients’ inputs and risks overestimating the benefits of a potentially endless array of health care interventions.

The practice of disregarding the time spent seeking health care may be particularly detrimental to women. As with other elements of care work in sub-Saharan Africa, the responsibility for seeking health care falls disproportionately on women [6–8]. This is partly due to biological differences that shape the health care needs of men and women, particularly around pregnancy and delivery. Yet, other routine health services that could be shared by men, such as family planning and children’s under-five visits, also fall heavily on women.

The time spent seeking health care includes travel to and from clinics, wait times for consultations, the consultation itself, and repeat visits (including all of the previously-mentioned factors) when providers or medications are not immediately available. In low-resource settings such as Malawi, the time required for all components of a health care visit is likely to be large: clients often travel long distances to reach health facilities; many services are not offered all day or every day; and, shortages of health care workers and drugs increase wait times and necessitate repeat visits [9, 10]. To some extent, waiting for services is a part of life in low-resource settings where services are scarce and absenteeism among service providers is common. Extended wait times may also be an accepted expression of local power hierarchies wherein those who seek care are at the mercy of those who offer it [11, 12]. Nonetheless, the time required for health care presents a major barrier to accessing and using it [5, 13, 14]. Apart from a few disease- or clinic-specific studies [1, 2, 15], however, there has been little systematic estimation of this time in a sub-Saharan African setting.

In this paper, we use population-based data from a cohort in southern Malawi to calculate the time young men and women spend seeking health care over a two-month period. We disaggregate findings by men, recently-pregnant women, mothers with children under two years old, and “other women”. We then extrapolate the time required for specific services to estimate the time that would be needed for each subpopulation to meet government recommendations for routine health services over the course of a year. Finally, we discuss the implications of the findings in light of women’s existing levels of time poverty and men’s poor health outcomes and offer a series of recommendations.

### Malawian context

Malawian women begin childbearing at a relatively early age (median age at first birth is 19) and have an average of 4.4 children per woman (a decrease from 5.7 in 2010) [16]. Women are less likely than men to be employed in the formal economy (63% vs. 81%) and less likely to complete secondary school (26% vs. 36%) [16]. Many individuals travel by foot to health clinics, and when possible, pay or borrow resources for transportation via bicycle or mini-bus. There has been a dramatic decentralization of health services over the past decade, but the distance to a clinic remains a primary barrier to care [16]. Most health services in Malawi (60%) are offered through government hospitals and clinics that are nominally free; the remainder of care is offered through mission hospitals and private clinics (40%) that require payment [17]. HIV remains a serious problem in Malawi and, with tuberculosis, is the primary cause of mortality. Among young adults ages 20–29, women are more than twice as likely to be infected with HIV as compared to men of the same age category [18]. Other major causes of morbidity and mortality include maternal health issues, malaria, respiratory illness, malnutrition and diarrheal diseases [19].

The Malawi government’s recommendations for routine health care are described in Table 1. The vast majority of recommended routine health visits target women of reproductive age and their children, a pattern that is not unique to Malawi and mirrors the priorities of overseas development assistance [20]. Although under-five recommendations are not by necessity the responsibility of mothers, norms in Malawi and elsewhere strongly reinforce that children’s health care is women’s work. There are no comparable guidelines for reproductive-age men, apart from medical male circumcision, a once in a lifetime procedure recommended to reduce HIV acquisition. The government recommends that both men and women get tested for HIV (HTC) annually or use antiretroviral treatment (ART) if already known to be HIVpositive. At the time of data collection, HIV-positive pregnant and breastfeeding women could initiate antiretroviral therapy (ART) immediately and for life (i.e., Option B+), while other adult populations had to meet WHO staging or CD4 count eligibility criteria before initiation [21]. Malawi has since shifted to a universal test and treat approach, under which all HIV-positive persons can initiate ART immediately and for life [22].

**Table 1:** Malawi government recommended health care guidelines and estimated annual time required to meet them.

	Service	Frequency of visits recommended	Target population	Median visit time^a^ (hours)	Estimated annual time required (hours)
Routine	Antenatal care (ANC)	4 visits during pregnancy	Pregnant women	4.7 (2.8–7.0)	18.7
	Delivery	1 visit at birth [19]	Pregnant women	24.8 (4.2–72.3)	24.8
	Post-natal care	1 visit at 6-weeks postpartum	Postpartum women	3.5 (2.3–6.0)	3.5
	Children: under-5 visit	1 visit per month for children 0–5 [23]	Parents	3.0 (1.8–5.0)	36.0
	Family planning	4–12 times a year, depending on the method used [24]	Women of reproductive age	4.0 (2.5–6.0)	16.0 to 48.0^b^
	HIV testing and counseling (HTC)	Every year and at the first ANC visit [25]	HIV-/Unknown	2.6 (1.3–4.6)	2.6
	Antiretroviral therapy	Every month for first 6 months, every 3 months thereafter [22]	All HIV+ persons^c^	5.0 (4.0–7.0)	20.0 to 40.0
Acute	Sickness^d^	Promptly, as needed	Men, women, and children	4.0 (2.3–6.5)	0.0 to 144.0^e^

Notes:

^a^Visit time refers to combined time spent at clinic and for return travel. Interquartile range (IQR) is in parentheses.

^b^Annual estimates are based on 4 visits, as this most closely aligns with the frequency required for injectable contraceptives, which comprise 50% of modern method use [16].

^c^In the first 6 months, new ART users should visit the clinic monthly to refill medication and be assessed for signs of treatment failure. Subsequently, appointments can extend to every 3 months at providers’ discretion. Annual estimates for meeting routine guidelines assume quarterly visits after the first 6 months, for a total of 8 visits during the first year on ART, and 4 visits per year thereafter.

^d^This includes malaria, STI, dental, and other acute care services.

^e^Based on range of 0 to 6 acute visits within a two-month time period among the TLT sample.

## Methods

We used data from Tsogolo la Thanzi (TLT), a 2009–2015 study of young adults in southern Malawi, to estimate the time men and women spend seeking health care for themselves and for their children. The original sample was drawn in 2009 to be representative of the population 15–25 years old living within seven kilometers of the Balaka market. The sample thus consists of a mix of urban residents living in town and residents of rural villages outside of town. The response rate of the original sample was 96% for women and 94% for men. The specific data we used come from the 2015 round of TLT in which 1453 women and 407 men aged 21–31 completed interviews (20% and 29% attrition, respectively). Trained TLT interviewers conducted the interviews face-to-face in Chichewa in private rooms at a central research center.

The TLT-2015 questionnaire included a module focused on all health services used over the previous two months. Respondents were asked to report on each visit made to a health facility or health care provider, including reason(s) for the visit, who received care (themselves, their children, or both), the one-way travel time (which we doubled to account for return travel) and the time it took to receive services, including waiting time (“clinic time”). Clinic and travel time were recorded in either minutes or hours, but we converted all durations to hours for analysis and combined clinic and travel time to estimate the total duration of a visit.

There were 10 pre-coded health service types: HTC, ART, family planning, antenatal care (ANC), labor and delivery, post-natal care (PNC), under-five care, sexually transmitted infection (STI), malaria, and dental. Respondents could also report an “other” category, and we re-categorized these responses into the pre-coded service types when appropriate. The remaining “other” service reasons were predominantly acute care such as diarrhea, coughing, or fever.

We distinguished routine care visits from acute care visits because the latter are made in response to symptoms that require immediate care, whereas the former are intended to prevent or moderate symptoms via recurring care. In Table 1, routine care visits include all those recommended for healthy pregnancy (i.e., ANC, delivery, PNC), under-five visits, and family planning. HIV services are also routine because HIV testing is recommended annually and regular ART visits are important for maintaining health and viral load suppression. Circumcision was excluded from estimates in Table 1 because it is a once in a lifetime event that, in Malawi, commonly occurs before adulthood, but the few reported visits for circumcision were included in other estimates of time spent seeking health care.

We stratified our analyses by gender. Given the heavy health care needs during pregnancy and early childhood, we disaggregated women based on whether they were pregnant or had young children. We categorized women as “recently-pregnant” if they were currently pregnant or had delivered within two months of the survey. We categorized women as “mothers of children under two years” if they reported having a child between 3 and 24 months old, capturing the most intensive period for under-five health services. Nine women fit both categories and were retained in the pregnant category. We categorized the remaining women as “other women”. We did not disaggregate men by the age of their children or partners’ pregnancy status because service use and recommendations do not vary for men.

For each subpopulation, we calculated the mean and median number of each of the following: visits, time spent seeking health services (per visit and over the two-month period), and time required for each routine service type using a “per person, per service type” approach. We privileged median time estimates because they avoid influence from outliers such as particularly long visits (e.g., labor and delivery) or inpatient services, which has the effect of biasing our estimates downward. We included visits in which more than one service was received in our calculations of total time spent seeking health care but not in our estimations of the length of specific services due to difficulty separating out the clinic time taken for each service. There is one exception: given that provider-initiated HIV testing is a routine part of a woman’s first ANC visit in Malawi, we consider combined ANC-HTC visits to be part of ANC [26].

Finally, we applied the estimates from the TLT study to the national health care guidelines to estimate the amount of time that would be required annually to meet the recommendations for each subpopulation.

All analyses were conducted using STATA version 14.2. We used Mann-Whitney (Wilcoxon) rank-sum tests to assess differences in the median time spent seeking health care by gender and subpopulation, chi-squared tests to test for differences in proportions, and independent two-sample t-tests to assess differences in mean number of health care visits and time spent on health care.

### Ethics

Ethical approval was granted by the Malawi National Health Sciences Research Committee and the Social and Behavioral Sciences Institutional Review Board at the University of Chicago. Respondents gave written consent to participate in TLT at recruitment and before each survey.

## Results

Table 2 presents key characteristics of the TLT sample in 2015 by gender. Men and women were between the ages of 21 and 31, which represents a key period for economic productivity and reproduction. Consistent with gender-specific patterns across Malawi^18^, women were more likely to be married (72% vs. 46%) and have children (85% vs. 50%), to be diagnosed as HIV positive (15% vs. 3%), and to be on ART if HIV positive (56% vs. 8%) than were similarly-aged men (p<0.001 for all comparisons).

**Table 2:** Sociodemographic characteristics of TLT sample in 2015 by subpopulation.

	Men	Women			
	Total	Total	Pregnant/recent delivery	Mothers of children <2	Other women
	*N* (%) or Mean (SD)				
*N*	407	1453	173	458	822
**Age, mean (SD)**	25.1 (3.1)	25.6 (3.3)	24.9 (3.2)	25.5 (3.2)	25.8 (3.3)
**Marital status**					
Married	185 (46%)	1048 (72%)	155 (89.6%)	390 (85.2%)	503 (61.2%)
Formerly Married	15 (4%)	174 (12%)	12 (6.9%)	46 (10.0%)	116 (14.1%)
Never Married	207 (51%)	231 (15%)	6 (3.5%)	22 (4.8%)	203 (24.7%)
**Childbearing experience**					
# living children, mean (SD)	0.88 (1.13)	1.95 (1.31)	1.88 (1.24)	2.50 (1.15)	1.66 (1.31)
**Education**					
No education	1 (0.3%)	13 (0.9%)	1 (0.6%)	5 (1.1%)	7 (0.9%)
Some primary	170 (41.8%)	837 (57.6%)	112 (64.7%)	305 (66.6%)	420 (51.1%)
Some secondary	206 (50.6%)	563 (38.8%)	56 (32.4%)	144 (31.4%)	363 (44.2%)
Some tertiary	30 (7.4%)	40 (2.8%)	4 (2.3%)	4 (0.9%)	32 (3.9%)
**Employment**					
No work	84 (21.0%)	692 (47.6%)	101 (58.4%)	221 (48.3%)	370 (45.0%)
Piece work	86 (21.1%)	88 (6.1%)	8 (4.6%)	26 (5.7%)	54 (6.6%)
Temporary employment	49 (12.0%)	141 (9.7%)	10 (5.8%)	40 (8.7%)	91 (11.1%)
Steady job	188 (46.2%)	532 (36.6%)	54 (31.2%)	171 (37.3%)	307 (37.4%)
**HIV status**					
HIV+ on ART	1 (0.3%)	120 (8.3%)	13 (7.5%)	41 (9.0%)	66 (8.0%)
HIV+ no ART	11 (2.7%)	94 (6.5%)	7 (4.1%)	11 (2.4%)	76 (9.3%)
HIV-/unknown	395 (97.1%)	1239 (85.3%)	153 (88.4%)	406 (88.7%)	680 (82.7%)

SD = standard deviation

**Fig 1:**
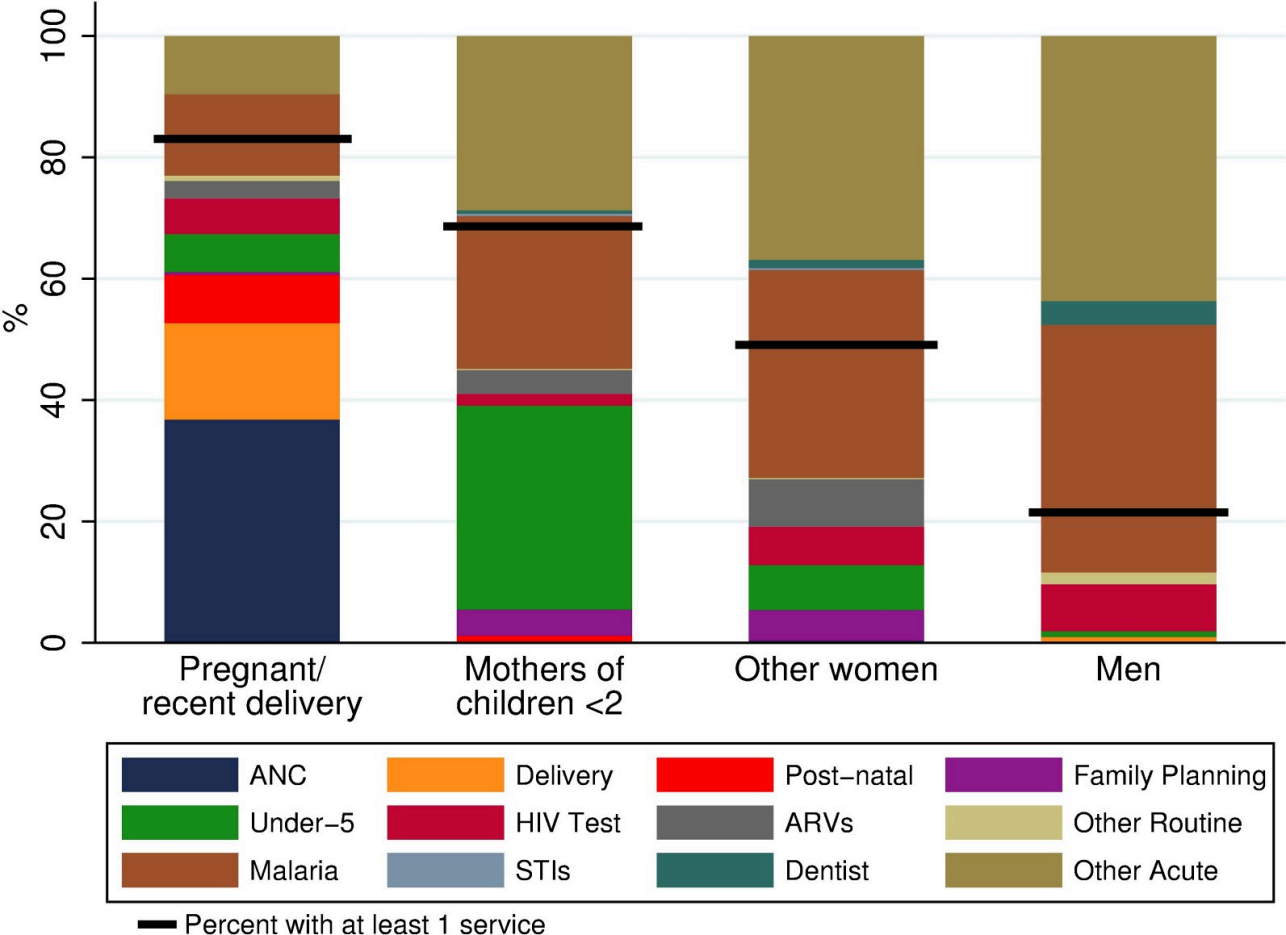
Main service types by subpopulation. Calculated as the percent of all services used by a subpopulation in a 2-month period.

Only 22% of men accessed health care during the two-month reference period. In contrast, 60% of women reported at least one health visit: 84% of recently-pregnant women, 70% of mothers of children under two, and 50% of “other women” (Table 3). Most (89%) of men’s visits were for acute care such as coughing or malaria (Fig 1). In contrast, women’s visits were largely for routine health care needs such as pregnancy-related care or under-five visits. The services used by “other women” were most similar to those used by men– 75% of their visits were for acute care, followed by a variety of routine services, including family planning, ART, and HTC. Fewer than 4% of all visits were for more than one service, reflecting the low-level of integrated care in Malawi. It was common for women to report multiple visits over the two-month period, with some reporting as many as six. On average, women reported 0.83 visits in two months vs. men’s 0.24 (t = -13.303, p<0.001) (Table 3). Recently-pregnant women and mothers of children under two years had the highest number of health care visits (1.20 and 1.04 visits, respectively). “Other women” had fewer visits but still more than men (0.63 vs. 0.24 visits, t = -9.461, p<0.001).

**Table 3:** Health service use over a 2-month period and associated time by subpopulation, TLT 2015.

	Men	Women			
	Total	Total	Pregnant/ recent delivery	Mothers of children <2	Other women
*N*	407	1453	173	458	822
**Health care use**					
Any visit, *N* (%)	90 (22.1%)	874 (60.2%)	145 (83.8%)	320 (69.9%)	409 (49.8%)
Any routine visit, *N* (%)	11 (2.7%)	406 (27.9%)	125 (72.3%)	159 (34.7%)	122 (14.8%)
Any acute visit, *N* (%)	80 (19.7%)	594 (40.9%)	47 (27.2%)	223 (48.7%)	324 (39.4%)
Mean # visits (SD, range)	0.24 (0.49, 0–3)	0.83 (0.85, 0–6)	1.20 (0.80, 0–4)	1.04 (0.93, 0–4)	0.63 (0.75, 0–6)
**Total time burden (hours)**					
Median (IQR)	0 (0–0)	2.2 (0–6.0)	5.0 (2.0–10.0)	3.5 (0–7.0)	0 (0–4.7)
Mean (SD, range)	1.0 (3.5, 0–54)	6.4 (24.6, 0–506)	15.2 (35.3, 0–310)	6.9 (24.6, 0–445)	4.2 (21.3, 0–506)
**Visit length (hours)**					
Total Time (median, IQR)	3.0 (2.0–4.7)	4.0 (2.5–6.5)	5.0 (3.0–8.4)	4.0 (2.5–6.3)	4.0 (2.3–6.0)
Travel Time (median, IQR)	1.0 (0.7–2.0)	2.0 (1.0–4.0)	2.0 (1.0–2.0)	2.0 (1.0–4.0)	2.0 (1.0–4.0)
Clinic Time (median, IQR)	1.0 (0.7–2.0)	2.0 (1.0–3.0)	2.0 (1.0–4.0)	2.0 (1.0–3.0)	1.5 (1.0–3.0)

SD = standard deviation; IQR = interquartile range

Note: Total time burden is the combination of time spent at clinic and for return travel in the 2-month period. Median visit lengths are calculated based on a per person average visit among those with at least one health service in the 2-month period.

Visits were uniformly long; however, women’s visits took longer than men’s. The median time spent at a clinic per visit for women was twice as long as that for men (2.0 hours vs. 1.0; z = -1.977, p = 0.048). The median travel time per visit to and from the clinic for women was also 2.0 hours, again twice that reported by men (z = -4.497, p<0.001). This may be explained, in part, by men’s greater access to bicycles (56% of men owned a bicycle vs. 14% of women). Apart from delivery, the longest visits, with clinic and travel time combined, were for ART and ANC, which took a median of 5.0 and 4.7 hours, respectively (Table 1).

**Fig 2:**
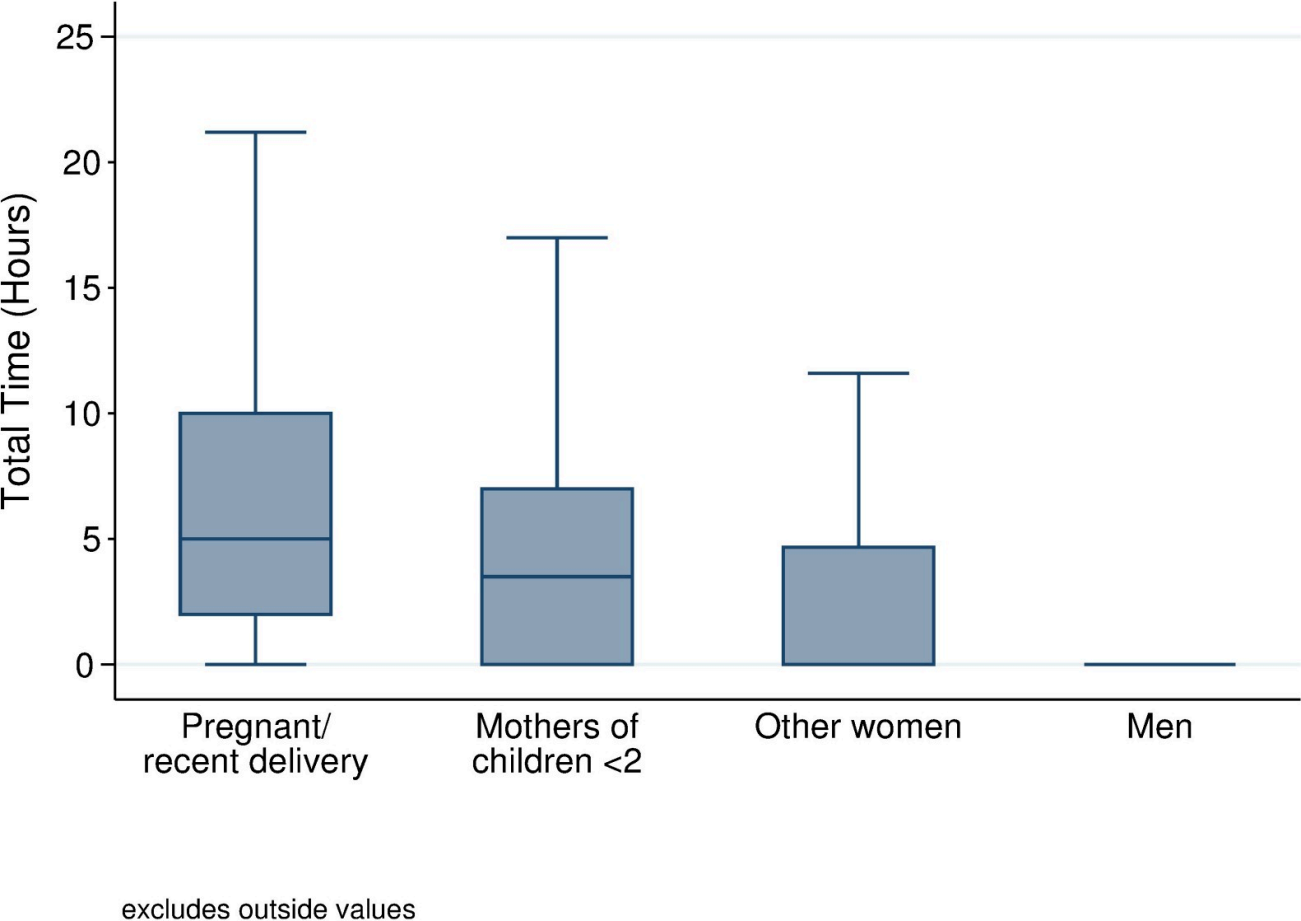
Total time burden of health care seeking over a two-month period by subpopulation. Values are excluded that fall outside 1.5 times the interquartile range [27].

Gender differences in number of visits and length of visits led to large aggregate differences in the total time men and women spent seeking health care over the two-month period (Table 3). Fig 2 illustrates the substantial variation in the total time burden for seeking care both across and within subgroups. Recently-pregnant women spent a median of 5.0 hours seeking care, while mothers of children under two spent 3.5 hours (z = -3.956, p<0.001). Although the median time burden for men and “other women” was 0 hours, the subgroup medians mask considerable variation among these two groups that is revealed in both the box plot and the means. “Other women” had a mean of 4.2 hours compared to men’s average of 1.0 hour (t = -3.038, p = 0.002). In total, women spent a mean of 6.4 hours seeking care over the period.

**Fig 3:**
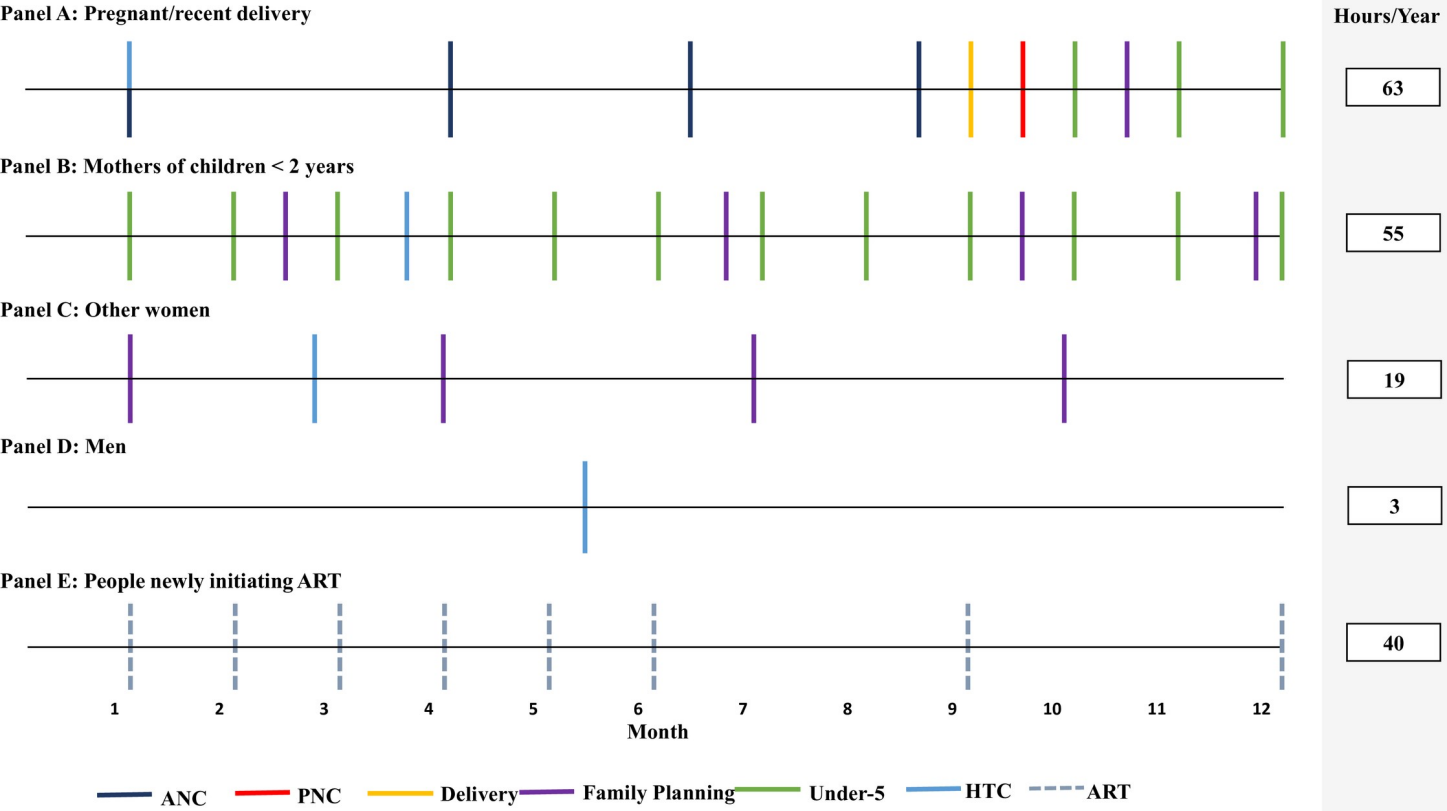
Annual recommended frequency of services and estimated time required by subpopulation. Visit frequency estimated based on national guidelines. Annual time required to meet guidelines is estimated from TLT calculations of median time required per service type (Table 1).

We applied the TLT service-specific estimates to the national guidelines to estimate the annual time required to meet each recommendation (Table 1). Fig 3 illustrates the frequency of recommended health care visits over a one-year period, and the time that would be required to complete these visits. There are large disparities in time requirements between subpopulations, which are driven not only by the length of each visit but also by the sheer number of visits that are recommended for certain groups, particularly pregnant women and mothers of children under two. As an illustration, an HIV-negative mother of children under two who wishes to delay a subsequent pregnancy would be expected to visit the clinic 17 times for routine care over a 12-month period, for a total of 55 hours or the equivalent of almost seven eight-hour work days (Fig 3, Panel B). If she were newly diagnosed as HIV positive, she would require another eight visits to the ART clinic, adding 40 hours (or five 8-hour working days) to her existing time burden. An HIV-negative woman who is neither pregnant nor has young children under two is expected to attend five routine health care visits, primarily for family planning, and would spend 19 hours seeking care. In contrast, an HIV-negative man in the same age-category would have only a single three-hour visit for HTC over the course of the year.

## Discussion

We found significant gender differences in health care seeking behavior among young women and men in southern Malawi. Young women were almost three times as likely as young men in our sample to use health services over a 2-month period and their median visit length was 33% longer. Based on these differences, women spent six times the total number of hours seeking care as did men, with a mean of 6.4 hours compared to 1.0 hour over the 2-month period (t = -4.414, p<0.001). The greatest time burden was experienced by recently-pregnant women and those with children under two years old. Nevertheless, women who were neither pregnant nor had young children still spent four times as long seeking health care as men.

Gender differences in seeking health care are largely shaped by Malawi’s national guidelines that call for numerous routine health visits for women, but almost none for men. Most recommendations for women are based on the unique reproductive health needs of women and the young children they care for. Use of these services is associated with improved maternal and child health outcomes [28, 29], which is critical in a context of relatively high maternal and child mortality such as Malawi [16]. However, services are rarely integrated, meaning that each recommendation is often associated with a seperate visit to the health facility. The siloed nature of services means the numerous recommendations translate to substantial time commitments from women. Applying median time estimates to national recommendations, we found that HIV-negative women of reproductive age are expected to visit a clinic at least quarterly and often more than once a month, requiring between 19 and 63 hours for health care each year compared to only 3 hours for HIV-negative men. Of course, guidelines are not always followed (in Malawi or elsewhere) [16, 30], but they remain important because they frame expectations for health seeking behavior and signal to communities the populations that are prioritized by health programs.

These findings may not surprise people acquainted with health services in low-resource contexts. Yet they should be concerning for at least three reasons. First, the time required to access health services is an important barrier to health care utilization [1, 5, 14, 31]. The low integration of health services results in high time burdens for women, and may mean women have to choose certain routine services over others.

Second, the extensive time required for health care seeking behavior can further exacerbate women’s time poverty [6, 32, 33]. Across sub-Saharan Africa, women’s work is frequently unpaid and goes largely unacknowledged [7, 34]. Health care seeking behavior is a form of unpaid labor that falls disproportionally on women, while the health improvements and downstream socioeconomic benefits resulting therefrom are shared by families and society. The recurring time burden associated with health care seeking can create competing demands between health care and women’s other responsibilities, and ultimately constrain women’s opportunities for education, paid and other unpaid labor, and leisure [35–38].

Third, men’s relative absence from health services contributes to high rates of male morbidity and mortality [39, 40]. In Malawi, and globally, men have a greater burden of disease and lower life expectancy than women [41]. Although men are often blamed for their absence from health facilities [42, 43], Malawi’s routine health care guidelines illustrate that national and global policies (Malawi’s health sector is funded in large part by international donors [44]) are largely silent to men’s health needs, except in response to acute illness and HIV testing [45–47]. This silence has historical origins in selective primary health care and the GOBI-FFF programming that centered on women and children’s health and included little if any services for men [48]. By excluding men from national guidelines and global policies, the public health community perpetuates the view that health care is for women and contributes to men’s socialization that they should avoid health facilities [42, 45, 49].

Several strategies may address the large gender disparities in time spent seeking health care, and the negative outcomes that affect women’s time and men’s health. First and foremost, integrated health services should be promoted to minimize repeat visits to the clinic and thus cut down on the substantial time women spend traveling to and from and clinics [50]. This is particularly critical for services frequented by women, such as family planning, other reproductive heatlh services, and services for children-under 5 years. Integrating services has proven challenging in resource-constrained settings [51, 52]; however, the cost-benefit calculus may change when the full cost of women’s time is considered. In settings where integration is not possible, services should be offered on a daily basis to minimize the number of visits required for women to meet basic health care recommendations.

Second, health outreach programs for under-five care and family planning services could reduce women’s time burden for health care seeking, while extending the reach of health services. Efficiencies are also possible within HIV care and treatment through the use of differentiated models of care for ART distribution, such as providing 3–6 months of drug distribution at each ART visit, fast-track drug refills, and community-based ART distribution strategies [53–55].

Third, family planning strategies should be prioritized. 15% of women in Malawi are estimated to have an unmet need for family planning [16]. Empowering women to manage their own fertility will dramatically reduce the amount of time women spend seeking pregnancy-related health care. However, the predominant contraceptive methods in Malawi (e.g., 3-monthly contraceptive injections) require frequent and lengthy visits to health clinics. Use of longer-acting reversible and non-reversible contraceptives has been increasing in the country [16], and continued expansion of these contraceptives where desired by women would reduce the burden of time required to prevent unintended pregnancies.

Finally, additional strategies are needed to engage men in health care. This is an uphill battle as deep-seated gender norms (held by both local communities and broader health systems) paint men as external to the health system. A critical step to engaging men is to introduce policies that provide universal entry points for men’s care, outside of acute illness or injury. Pregnancy may provide an ideal universal entry point since men are already encouraged to attend antenatal services with their female partners to provide support for women and children’s health [56]. New strategies could provide “Family Health” services during pregnancy, whereby women receive antenatal services and men receive preventative health and screening services. Additional efforts are also needed to create male-friendly spaces within health facilities such as convenient and flexible service hours and waiting spaces designated for men [49]. Finally, changes to cultural norms require patience—normalizing men’s routine and frequent use of health services will take time and requires multi-level interventions within health systems and local communities. In the meantime, health care services must reach out to men where they are through community- and work-based health services, as has been done successfully with HIV testing [57].

Our results are subject to limitations. We relied on retrospective estimates that are susceptible to telescoping and recall bias. Additionally, although women are more likely to accompany other family members to clinics [58, 59], these visits were not captured in the TLT survey. This suggests the amount of time women spend seeking care is even greater than our estimates indicate. TLT also lacked data on men’s visits to ANC; however, in practice, partner attendance at ANC remains low (women reported their male partner accompanied them to ANC at 16% of such visits). Certain acute health visits (e.g., malaria) and travel times vary by season. TLT collected data over a four-month period (mostly within the dry season) which may have resulted in underestimates of our 12-month extrapolations. Lastly, our data come from a single setting in southern Malawi, and travel times and clinic wait times will vary across the country. However, the mix of town residents and rural villagers in the TLT sample increase the likelihood that our estimates reflect a common Malawian experience and the overall patterns we identified are likely to be similar elsewhere. Indeed, our estimates of the mean time required for HTC were similar to those from a recent study based elsewhere in Malawi (e.g., a mean of 3.5 hours vs. 3.2 hours for women in the TLT study site) [60].

Young women in Malawi spend large amounts of time seeking health care, while men are largely absent from the health care system. New strategies are needed to promote health and decrease time poverty for both women and men. Until we acknowledge the unpaid health care work that women do in service of their families and communities, and create efficiencies to minimize this time, we risk inadvertently limiting women’s participation in other sectors and devaluing the vast contribution women make to population health. Conversely, until we successfully engage men within the health care system we will continue to see disporportionate rates of male morbidity and mortality across a range of illnesses.

## Supporting information

S1 File STROBE checklist. (DOC)
