# Supplementary material for: Women's (health) work: A population-based, cross-sectional study of gender differences in time spent seeking health care in Malawi
Source: PLoS One. 2018 Dec 21;13(12):e0209586. doi: 10.1371/journal.pone.0209586 (PMC6303093; doi:10.1371/journal.pone.0209586)
Supplement: S1 File — (DOC) [file pone.0209586.s001.doc]

STROBE Statement—Checklist of items that should be included in reports of ***cross-sectional studies***

|  | Completion | Recommendation | |
| --- | --- | --- | --- |
| **Title and abstract** | (a)Women’s (health) work: a population-based, cross-sectional study of gender differences in time spent seeking health care in Malawi  (b)Please see the abstract for the text that includes a full and balanced summary of our methods and findings. | (*a*) Indicate the study’s design with a commonly used term in the title or the abstract | |
|  |  | (*b*) Provide in the abstract an informative and balanced summary of what was done and what was found | |
| Introduction | | |  |
| Background/rationale | The background and rational are described on page 3 in paragraph 1 of the Introduction section. | Explain the scientific background and rationale for the investigation being reported | |
| Objectives | The specific aims of the study are stated on page 4, in paragraph 4 of the Introduction section. | State specific objectives, including any prespecified hypotheses | |
| Methods | | |  |
| Study design | We conducted cross-sectional observational analyses of the 2015 wave of the Tsogolo la Thanzi study. This is presented on page 7, in paragraph 1 of the Methods section. | Present key elements of study design early in the paper | |
| Setting | The setting is described on page 7, in paragraph 1 of the Methods section. | Describe the setting, locations, and relevant dates, including periods of recruitment, exposure, follow-up, and data collection | |
| Participants | Study participants are described on page 7, in paragraph 1 of the Methods section. | (*a*) Give the eligibility criteria, and the sources and methods of selection of participants | |
| Variables | All variables are defined on pages 7 and 8, in paragraphs 2 and 3 of the Methods section. | Clearly define all outcomes, exposures, predictors, potential confounders, and effect modifiers. Give diagnostic criteria, if applicable | |
| Data sources/ measurement | All data come from the same survey. Measures are described on pages 7-9 of the Methods section. The same measures were collected from all respondents. | For each variable of interest, give sources of data and details of methods of assessment (measurement). Describe comparability of assessment methods if there is more than one group | |
| Bias | On page 8, in paragraph 6 of the Methods section, we discuss our handling of measures, and privileging of medians over means, as a way to reduce bias. | Describe any efforts to address potential sources of bias | |
| Study size | All respondents from existing secondary data from a population cohort were used. | Explain how the study size was arrived at | |
| Quantitative variables | Descriptions of how we handled number of visits and time spent at each visit are reported on page 7 in paragraph 2 and page 8 in paragraph 5 of the Methods section. | Explain how quantitative variables were handled in the analyses. If applicable, describe which groupings were chosen and why | |
| Statistical methods | (a)The statistical methods are described on pages 8 in paragraph 6 and page 9 in paragraph 8 of the Methods section.  (b)Subgroup analyses are described on page 8, paragraph 4 of the Methods section. Data were analysed for four subgroups: men, women who were pregnant or recently delivered, women who were mothers of children under two years of age and “other” women.  (c)There were no missing data on time spent seeking health care and only two missing values on employment for men. These were filled in using data from elsewhere in the questionnaire. (d)The TLT sample was drawn as a simple random sample of women and of men 15-25 (in 2009) living within a 7-km radius of Balaka, Malawi. Women were oversampled; however, since the data are stratified by gender throughout, no steps were needed to account for this.  (e)None. The largely descriptive nature of the study did not necessitate sensitivity analyses. | (*a*) Describe all statistical methods, including those used to control for confounding | |
|  |  | (*b*) Describe any methods used to examine subgroups and interactions | |
|  |  | (*c*) Explain how missing data were addressed | |
|  |  | (*d*) If applicable, describe analytical methods taking account of sampling strategy | |
|  |  | (*e*) Describe any sensitivity analyses | |
| Results | | |  |
| Participants | (a)Data come from the full sample of 1453 women and 407 men interviewed in 2015. We report on levels of attrition from the original 2009 sample (page 7, paragraph 1 of the Methods section).  (b) Not applicable  (c) Not applicable | (a) Report numbers of individuals at each stage of study—eg numbers potentially eligible, examined for eligibility, confirmed eligible, included in the study, completing follow-up, and analysed | |
|  |  | (b) Give reasons for non-participation at each stage | |
|  |  | (c) Consider use of a flow diagram | |
| Descriptive data | (a)The characteristics of the study participants are presented in Table 2 and discussed on page 9, paragraph 1 of the Results section.  (b) As discussed above, there were no missing data on key variables. | (a) Give characteristics of study participants (eg demographic, clinical, social) and information on exposures and potential confounders | |
|  |  | (b) Indicate number of participants with missing data for each variable of interest | |
| Outcome data | These are reported in Tables 2 and 3 and throughout the Results section. | Report numbers of outcome events or summary measures | |
| Main results | (a) Our analysis is a rich descriptive analysis of something that has never previously been measured in the literature. Confounder-adjusted estimates are not applicable, although we consistently stratify our results by key subgroups.  (b) Not applicable  (c) Not applicable | (*a*) Give unadjusted estimates and, if applicable, confounder-adjusted estimates and their precision (eg, 95% confidence interval). Make clear which confounders were adjusted for and why they were included | |
|  |  |  |  |
|  |  | (*b*) Report category boundaries when continuous variables were categorized | |
|  |  | (*c*) If relevant, consider translating estimates of relative risk into absolute risk for a meaningful time period | |
| Other analyses | Not applicable. | Report other analyses done—eg analyses of subgroups and interactions, and sensitivity analyses | |
| Discussion | | |  |
| Key results | Key results are summarised on pages 15 and 16 in paragraph 1 of the Discussion section. | Summarise key results with reference to study objectives | |
| Limitations | The limitations of the study are discussed on page 18 in paragraph 8 of the Discussion section. | Discuss limitations of the study, taking into account sources of potential bias or imprecision. Discuss both direction and magnitude of any potential bias | |
| Interpretation | This occurs throughout the Discussion section (pages 15-18). | Give a cautious overall interpretation of results considering objectives, limitations, multiplicity of analyses, results from similar studies, and other relevant evidence | |
| Generalisability | The generalisability of the study is discussed on page 18 in paragraph 8 of the Discussion section. | Discuss the generalisability (external validity) of the study results | |
| Other information | | |  |
| Funding | Data collection and some of the authors’ time was funded by grants R01-HD058366 and R01-HD077873 from the U.S. National Institute of Child Health and Human Development. KD’s time is partially supported by the U.S. Agency for International Development (USAID) and the President’s Emergency Plan for AIDS Relief (PEPFAR) under Cooperative Agreement AID-OAA-A-15-00070 and the National Institute of Mental Health (NIMH) through T32MH080634-10. The funders had no role in study design; data collection, analysis, or interpretation; or writing of the article. | Give the source of funding and the role of the funders for the present study and, if applicable, for the original study on which the present article is based | |

*Give information separately for exposed and unexposed groups.

**Note:** An Explanation and Elaboration article discusses each checklist item and gives methodological background and published examples of transparent reporting. The STROBE checklist is best used in conjunction with this article (freely available on the Web sites of PLoS Medicine at http://www.plosmedicine.org/, Annals of Internal Medicine at http://www.annals.org/, and Epidemiology at http://www.epidem.com/). Information on the STROBE Initiative is available at www.strobe-statement.org.
